# Artificial intelligence-based prediction of organ involvement in Sjogren’s syndrome using labial gland biopsy whole-slide images

**DOI:** 10.1007/s10067-025-07518-5

**Published:** 2025-06-05

**Authors:** Yong Ren, Wenqi Xia, Jiayun Wu, Zheng Yang, Ye Jiang, Ya Wen, Qiuquan Guo, Jieruo Gu, Jun Yang, Jun Luo, Qing Lv

**Affiliations:** 1 University of Electronic Science and Technology of China, Chengdu, 611731 China; 2 Department of Rheumatology, The Seventh Affiliated Hospital of Sun Yat-Sen University, Shenzhen, 518107 China; 3 Department of Pathology, The Seventh Affiliated Hospital of Sun Yat-Sen, University, Shenzhen, 518107 China; 4 Department of Pathology, The Third Affiliated Hospital of Sun Yat-Sen, University, Guangzhou, 510630 China; 5 Department of Rheumatology, The Third Affiliated Hospital of Sun Yat-Sen University, Guangzhou, 510630 China; 6 ShenSi Lab, Shenzhen Institute for Advanced Study, University of Electronic Science and Technology of China, Shenzhen, 518038 China

**Keywords:** Deep learning, Organ involvement, Sjogren’s syndrome, Whole-slide images

## Abstract

**Objectives:**

This study aimed to develop a deep learning-based model to predict the risk of high-risk extra-glandular organ involvement (HR-OI) in patients with Sjogren’s syndrome (SS) using whole-slide images (WSI) from labial gland biopsies.

**Methods:**

We collected WSI data from 221 SS patients. Pre-trained models, including ResNet50, InceptionV3, and EfficientNet-B5, were employed to extract image features. A classification model was constructed using multi-instance learning and ensemble learning techniques.

**Results:**

The ensemble model achieved high area under the receiver operating characteristic (ROC) curve values on both internal and external validation sets, indicating strong predictive performance. Moreover, the model was able to identify key pathological features associated with the risk of HR-OI.

**Conclusions:**

This study demonstrates that a deep learning-based model can effectively predict the risk of HR-OI in SS patients, providing a novel basis for clinical decision-making.

**Table:** 

**Key Points** **1. What is already known on this topic?** • *Sjogren’s syndrome (SS) is a chronic autoimmune disease affecting the salivary and lacrimal glands.* • *Accurate prediction of high-risk extra-glandular organ involvement (HR-OI) is crucial for timely intervention and improved patient outcomes in SS.* • *Traditional methods for HR-OI prediction rely on clinical data and lack objectivity.* **2. What this study adds?** • *This study proposes a novel deep learning-based model using whole-slide images (WSI) from labial gland biopsies for predicting HR-OI in SS patients.* • *Our model utilizes pre-trained convolutional neural networks (CNNs) and a Vision Transformer (ViT) module to extract informative features from WSI data.* • *The ensemble model achieves high accuracy in predicting HR-OI, outperforming traditional methods.* • *The model can identify key pathological features in WSI data associated with HR-OI risk.* **3. How this study might affect research, practice or policy?** • *This study provides a novel and objective approach for predicting HR-OI in SS patients, potentially leading to improved clinical decision-making and personalized treatment strategies.* • *Our findings encourage further investigation into the role of deep learning and WSI analysis in SS diagnosis and risk stratification.* • *The development of a non-invasive and objective diagnostic tool based on WSI analysis could benefit clinical practice and inform policy decisions regarding patient care for SS.The development of a non-invasive and objective diagnostic tool based on WSI analysis could benefit clinical practice and inform policy decisions regarding patient care for SS.*

**Supplementary Information:**

The online version contains supplementary material available at 10.1007/s10067-025-07518-5.

## Introduction

Primary Sjogren’s syndrome (SS) is a systemic autoimmune disease with a prevalence of 0.01–0.09% in the general population, and its incidence has been on the rise [1, 2]. In addition to systemic manifestations such as fatigue, inflammation, and pain related to depression, which are similar to other diffuse connective tissue diseases, SS exhibits clinical heterogeneity [3, 4]. Approximately 89–98% of SS patients present with exocrine gland dysfunction (salivary glands and/or lacrimal glands), while 40–50% exhibit involvement of various vital organs, including interstitial pneumonia, renal tubular acidosis and renal insufficiency, central nervous system damage, immune thrombocytopenia, and vasculitis [5–8]. Involvement of vital organs is significantly associated with the prognosis of SS, directly reducing patients’ quality of life and being a major cause of death in SS patients [9, 10]. They also impose a significant economic burden on patients and their families. Recent studies have shown that factors such as age, disease duration, high titers of anti-La-SSB antibodies, and low complement levels may be associated with significant organ damage and disease prognosis in Sjogren’s syndrome [11, 12]. However, there is currently no definitive explanation for the differences in clinical manifestations (i.e., the presence or absence of extra-glandular major organ involvement) [13, 14]. Clinically, it is sometimes difficult to distinguish between these two types of patients, especially in the early stages of the disease. This makes it challenging to provide clear answers to patients regarding disease prognosis, follow-up monitoring frequency, and even certain drug choices. For some patients in the early stages of the disease, the golden opportunity for prevention and early intervention of severe HR-OI may even be missed [15, 16].

Labial gland histopathology plays a crucial role in the diagnosis and treatment of Sjogren’s syndrome. Focal lymphocytic aggregates and acinar atrophy in labial gland pathology have long been one of the important diagnostic criteria for Sjogren’s syndrome, and can also be used to differentiate diseases such as IgG4-related disease and lymphoma [17, 18]. The extent of gland involvement, including the range of lymphocytic infiltration and the degree of glandular tissue destruction, can help assess the disease activity. During treatment, dynamic observation of glandular pathological changes can help assess treatment efficacy and monitor disease progression (although we generally try to avoid repeated invasive examinations) [19]. However, our current understanding and application of traditional labial gland pathology have significant limitations. On the one hand, traditional labial gland biopsy only provides information about local inflammation and is currently used to assess local histopathological changes in the labial gland, such as lymphocytic infiltration, but cannot comprehensively reflect systemic disease activity, damage to organs and tissues outside the gland, and concurrent immune diseases [20]. On the other hand, traditional pathological reports mostly describe glandular inflammation, destruction, and atrophy qualitatively and semi-quantitatively, failing to fully utilize all the pathological feature information contained in the biopsy images, and it is difficult to provide accurate quantitative indicators to assess disease severity and the risk of disease prognosis-related manifestations [21]. Therefore, it is necessary to combine with other new technologies and develop new evaluation models to fully utilize histopathological information and improve the basis for disease diagnosis, treatment, and prognosis judgment.

In recent years, artificial intelligence (AI), especially deep learning (DL) technology, has developed by leaps and bounds, thanks to the rapid development of computer technology and the internet [22–24]. Digital pathology images (whole-slide image, WSI) are excellent materials for training and modeling AI based on deep learning. DL-based pathology diagnosis systems are realizing tasks that were previously only possible for humans, and have shown unique advantages in the field of pathology with abundant data [25–27]. DL-assisted pathology image analysis is quietly changing the traditional way of reading pathology slides, and it can improve the objectivity and accuracy of pathological diagnosis, and has been successfully applied in many fields, including the grading diagnosis and prognosis prediction of breast cancer and lung cancer [25, 28, 29].

Applying AI and digital pathology technology to evaluate the risk of high-risk extra-glandular organ involvement (HR-OI) in SS based on WSI from labial gland biopsies can overcome the subjectivity and qualitative description of traditional evaluation methods [30]. By using DL algorithms to extract and mine a large number of subtle pathological features that are difficult for the human eye to capture from WSI, it is possible to achieve accurate quantification and standardization of pathological indicators, and to construct a multi-modal individualized predictive model by integrating other clinical data, thereby significantly improving the prediction accuracy and interpretability of the risk of HR-OI [28, 29, 31]. In addition, AI-assisted diagnosis systems can help improve work efficiency, dynamically monitor risk changes, provide strong support for achieving individualized risk assessment and developing precise treatment plans, and have important clinical translation value for improving the prognosis and quality of life of patients with SS [28–32].

## Methods

### Study design

This retrospective cross-sectional study enrolled 221 consecutive patients with SS at their first hospital visit. The study objective was to distinguish patients who already had high-risk extra-glandular organ involvement (HR-OI) from those without HR-OI by analyzing a single time-point labial-salivary-gland WSI. All participants were treatment naive at the time of biopsy, drug exposure, and clinical response were not captured in this cross-sectional dataset. Patients were allocated exclusively to one of two mutually-exclusive strata: (i) HR-OI group – at least one extra-glandular major-organ involvement (lung, kidney, hematological system, or central nervous system), irrespective of concomitant glandular manifestations and (ii) low-risk group – no evidence of extra-glandular organ disease, i.e., glandular-only involvement. Patients with multiple organs affected were retained in the HR-OI stratum and each organ was recorded separately [10–14, 18–21].

### Data collection

Among these patients, 124 were treated at the Seventh Affiliated Hospital of Sun Yat-sen University from August 2020 to June 2023, forming dataset 1, which was used to construct the model training dataset and internal test set. The remaining 97 patients were treated at the Third Affiliated Hospital of Sun Yat-sen University from March 2014 to March 2019, forming dataset 2, which served as the external validation set for the model. The study was approved by the Ethics Committee of the Third Affiliated Hospital of Sun Yat-sen University. Since this was a retrospective study, no patients received any rewards or compensation for participating. Patient clinical data, systemic assessments, and pathological examination results were all obtained from the hospital information system.

### Whole-slide imaging workflow

Labial-salivary-gland biopsies were fixed, H&E-stained, and digitized at × 40 magnification on high-resolution scanners. Images passed routine quality control and were stored in a secure server. Each slide was automatically segmented into 512 × 512-pixel patches; empty or blurred patches were discarded [23–27] (detailed preprocessing parameters are provided in Supplementary Method).

### Feature encoding

For every retained patch, visual patterns were encoded with three complementary, ImageNet-pre-trained convolutional backbones (ResNet-50, Inception-V3, EfficientNet-B5) [22]. The resulting vectors serve as compact “fingerprints” of local histology (network architecture and training settings in Supplementary Method).

### Slide-level aggregation

Because prognostic information is distributed sparsely, we adopted a multi-instance learning strategy. A Vision Transformer summarised patch vectors into a single slide representation, highlighting regions most related to extra-glandular organ involvement (HR-OI).

### Ensemble risk score (RAIPSS)

Predictions from the three backbone-specific models were averaged to give a continuous HR-OI risk score (HR-OIRS, 0–1). A score ≥ 0.50 flagged patients at high risk for major-organ disease.

### Model explanation

Attention heat-maps overlayed on the original slide reveal histological foci driving each prediction, facilitating clinico-pathological correlation (example in Fig. 4).

### Statistical evaluation

Discrimination was assessed by AUC and F1 score. Accuracy, sensitivity, and specificity are reported in Tables 2 and 3. External validation was performed on an independent hospital cohort.

## Results

### Baseline characters

**Fig. 1:**
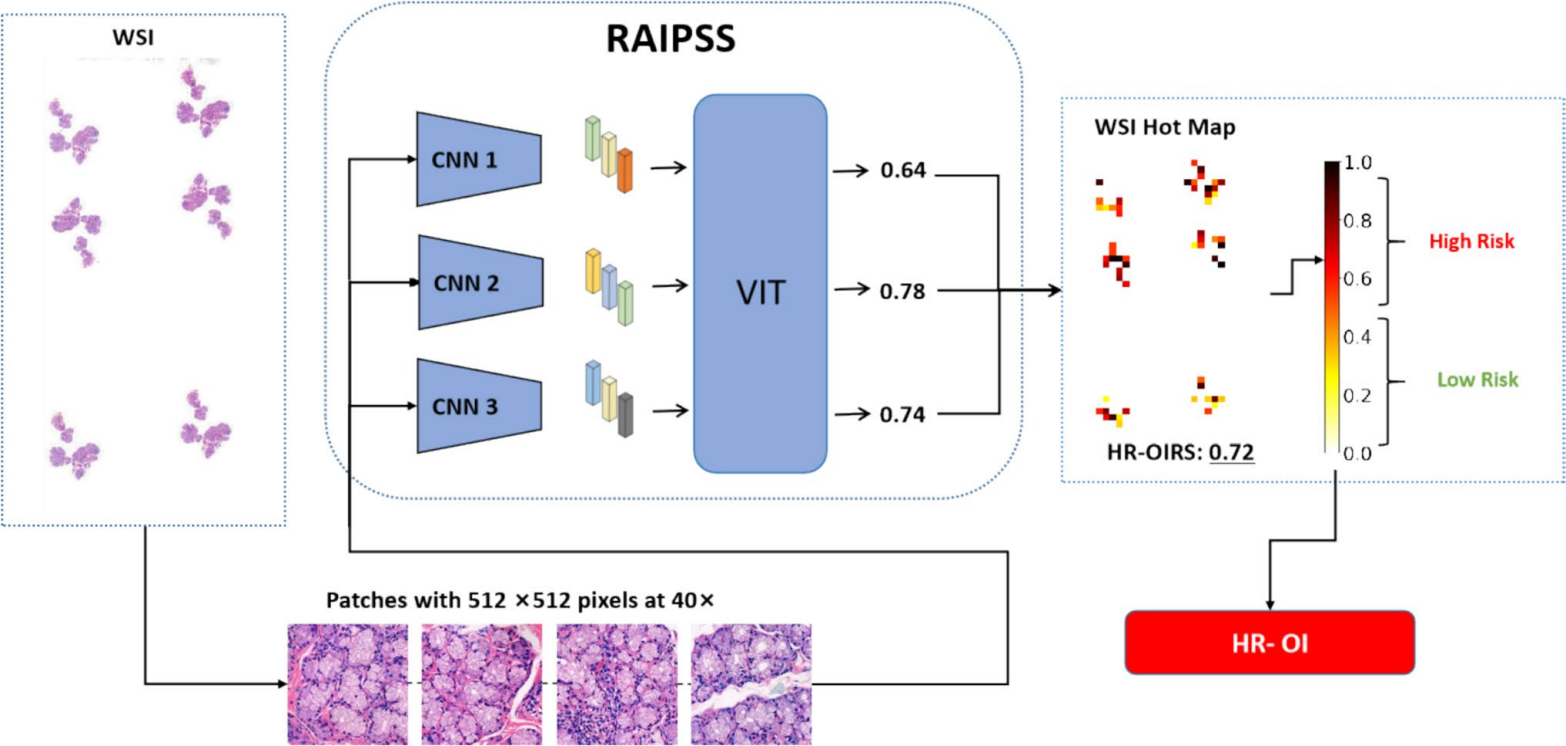
Research roadmap. Labial gland biopsy samples were collected from patients and scanned to obtain whole-slide images (WSIs). Subsequently, pre-processing was performed to generate 512 × 512 pixel patches. These patches were then fed into three convolutional neural networks (CNNs) and one Vision Transformer (ViT) module, respectively, to obtain three predicted probabilities of high-risk extra-glandular organ involvement (HR-OI). Finally, the three probabilities were averaged, and the ensemble model RAIPSS outputted the final risk score (HR-OIRS) and visualized heatmaps. If HR-OIRS exceeded 0.5, the model predicted a high risk of HR-OI in the patient

The baseline characteristics of the participants are shown in Table 1. In terms of demographics, the sex distribution in both cohorts matched the known epidemiology of SS—male/female ratios of 5/38 and 7/74 in the dataset 1 and 1/49 and 1/46 in the dataset 2—and the mean age across all subgroups was similar (44–48 years). With respect to organ involvement, hmatological manifestations were the most prominent in the high-risk group (41.9% in dataset 1 and 78.0% in dataset 2), markedly higher than the rates of pulmonary (12.0%) and renal (16.0%) involvement. Immunologically, serum IgG levels showed an upward trend in the high-risk group in both cohorts (dataset 1: 18.34 ± 18.28 g/L vs 15.39 ± 4.93 g/L; dataset 2: 21.32 ± 8.52 g/L vs 18.64 ± 6.79 g/L). Regarding inflammatory markers, ESR rose more markedly than CRP. Positivity rates for RF, anti-SSA, and anti-SSB antibodies were broadly similar between the two cohorts, with no significant difference between high- and low-risk strata. Importantly, none of the low-risk patients exhibited major-organ involvement, indicating that this stratification scheme discriminates clinical phenotypes well. RAIPSS, the proposed ensemble AI system, is shown in Fig. 1.

**Table 1:** Baseline characteristics of patients

	Dataset 1 (*n* = 124)		Dataset 1 (*n* = 97)	
	High-risk group (*n* = 43)	Low-risk group (*n* = 81)	High-risk group (*n* = 50)	Low-risk group (*n* = 47)
Demographics				
Gender(M/F)	5/38	7/74	1/49	1/46
Age(Y)	47.13 ± 14.83	48.09 ± 12.56	46.08 ± 8.97	44.30 ± 11.13
Organ involvement				
Pulmonary	37.21% (16/43)	0% (0/81)	12.00% (6/50)	0% (0/47)
Renal	25.58% (11/43)	0% (0/81)	16.00% (8/50)	0% (0/47)
Hematological	41.86% (18/43)	0% (0/81)	78.00% (39/50)	0% (0/47)
CNS*	7.98% (3/43)	0% (0/81)	12.00% (6/50)	0% (0/47)
Immunoglobulins and complements				
IgA	2.76 ± 1.07	2.62 ± 0.99	3.71 ± 3.06	3.14 ± 1.37
IgG	18.34 ± 18.28	15.39 ± 4.93	21.32 ± 8.52	18.64 ± 6.79
IgM	1.24 ± 0.93	1.12 ± 0.52	1.53 ± 1.24	1.45 ± 1.16
C3	0.93 ± 0.21	1.02 ± 0.21	1.04 ± 0.22	1.02 ± 2.23
C4	0.20 ± 0.07	0.22 ± 0.08	0.23 ± 0.09	0.23 ± 0.10
Inflammatory markers				
CRP	2.79 ± 3.63	5.07 ± 10.62	8.23 ± 25.71	5.48 ± 11.82
ESR	35.99 ± 27.17	31.49 ± 23.64	50.35 ± 38.93	34.71 ± 29.49
Autoantibody profile				
RF +	39.53% (17/43)	38.27% (31/81)	30.00% (15/50)	34.04% (16/47)
Anti-Ro/SSA +	72.09% (31/43)	58.02% (47/81)	90.00% (45/50)	74.46% (35/47)
Anti-La/SSB +	39.53% (17/43)	27.16% (22/81)	48.00% (24/50)	36.17% (17/47)

*CNS, central nervous system

### Model performance comparison

**Fig. 2:**
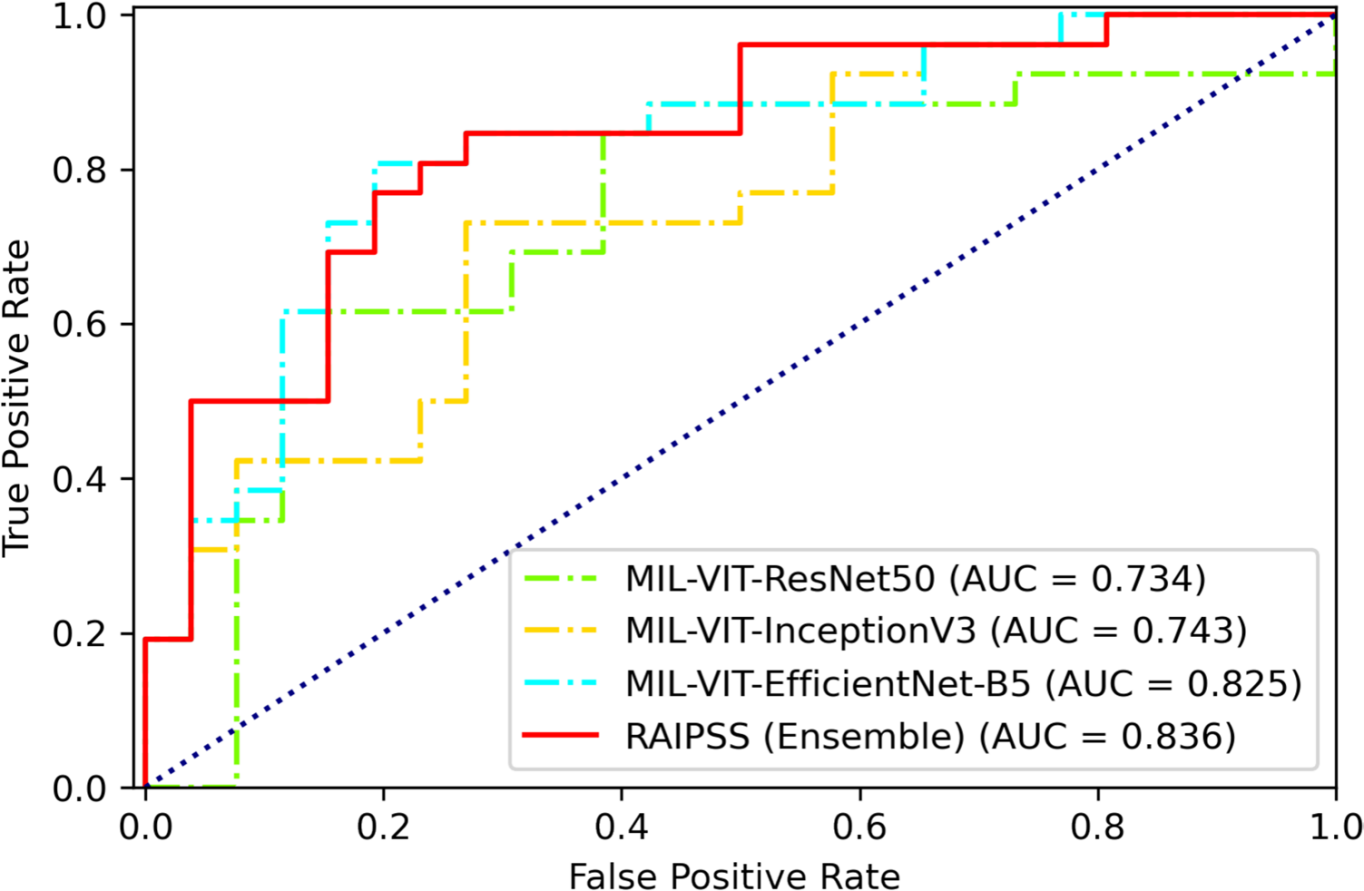
The receiver operating characteristic (ROC) curve of the models in predicting the HR-OI in SS on the internal test dataset

To evaluate the performance of the proposed models, we conducted a comprehensive comparison on the internal test set of dataset 1. Figure 2 depicts the receiver operating characteristic (ROC) curves of the four models: ResNet50, InceptionV3, EfficientNet-B5, and the ensemble model. Table 2 presents a detailed comparison of various performance metrics, including area under the ROC curve (AUC), accuracy, positive predictive value (PPV), negative predictive value (NPV), sensitivity (SENS), specificity (SPEC), and F1-score.

**Table 2:** Performance of the models in predicting the HR-OI in SS on the internal test dataset

Model	AUC	ACC	PPV	NPV	SENS	SPEC	F1
MIL (VIT-ResNet50)	0.734	0.70	0.62	0.77	0.73	0.67	0.69
MIL (VIT-InceptionV3)	0.743	0.75	0.64	0.83	0.82	0.67	0.73
MIL (VIT-EfficientNet-B5)	0.825	0.77	0.81	0.76	0.64	0.89	0.80
RAIPSS (ensemble)	0.836	0.80	0.79	0.78	0.70	0.87	0.81

*AUC*, area under the curve; *ACC*, accuracy; *PPV*, positive predictive value; *NPV*, negative predictive value; *SENS*, sensitivity; *SPEC*, specificity; *F1*, a weighted average of the PPV and SENS

The ensemble model, which combines the predictions of the three individual CNN-ViT models, consistently outperformed the individual models across all evaluation metrics. It achieved the highest AUC of 0.836, indicating its superior ability to discriminate between positive and negative cases. Additionally, the ensemble model exhibited high accuracy (0.80), PPV (0.79), NPV (0.78), sensitivity (0.70), specificity (0.87), and F1-score (0.81), demonstrating its robust performance in terms of both classification accuracy and balanced error rates.

In contrast, while ResNet50, InceptionV3, and EfficientNet-B5 achieved respectable performance, their individual AUC values were lower than that of the ensemble model, indicating a potential for improvement in their ability to discriminate between classes. The ensemble model’s improved performance can be attributed to the reduction of variance and the exploitation of complementary information from the individual models.

Based on these results, the ensemble model, which we have named RAIPSS, was selected as the optimal model for our task. The superior performance of RAIPSS underscores the effectiveness of our proposed approach in leveraging the strengths of multiple deep learning architectures to improve the accuracy and robustness of medical image analysis.

### External validation

**Fig. 3:**
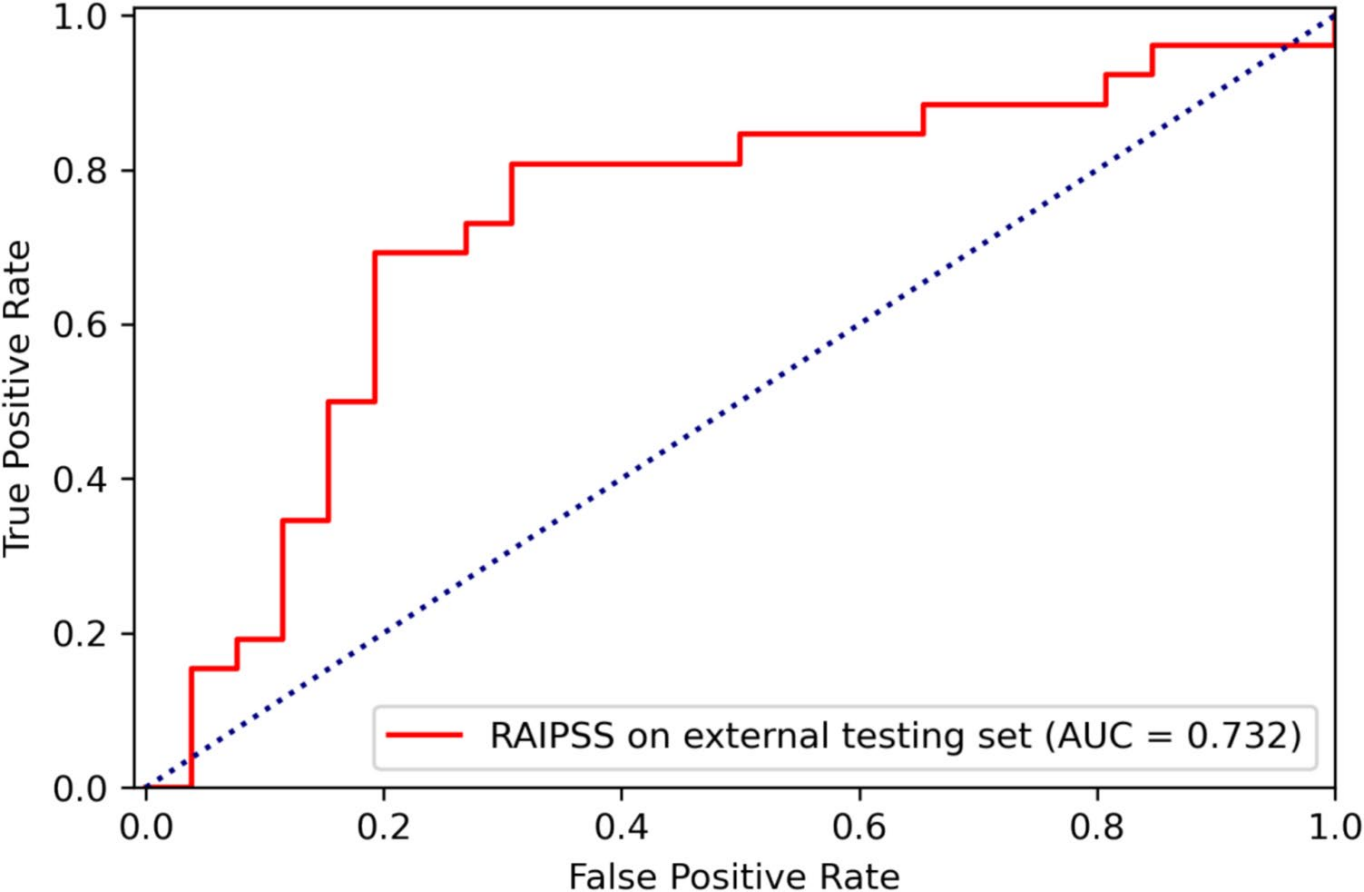
The receiver operating characteristic (ROC) curve of the RAIPSS in predicting the HR-OI in SS on the external test dataset

Deep learning algorithms are predisposed to overfit to the dataset they were trained on and thus must be validated with external datasets [24–28]. To assess the generalizability of the RAIPSS model, we conducted an external validation on an independent test set (dataset 2). Figure 3 and Table 3 present the performance metrics of RAIPSS on this external dataset. The obtained AUC, ACC, PPV, NPV, SENS, SPEC, and F1-score were 0.732, 0.74, 0.67, 0.79, 0.73, 0.73, and 0.73, respectively. Compared to the internal test set, a slight decrease was observed in most performance metrics, except for NPV and sensitivity.

**Table 3:** Performance of the RAIPSS in predicting the HR-OI in SS on the external test dataset

Model	AUC	ACC	PPV	NPV	SENS	SPEC	F1
RAIPSS (ensemble)	0.732	0.74	0.67	0.79	0.73	0.73	0.73

*AUC*, area under the curve; *ACC*, accuracy; *PPV*, positive predictive value; *NPV*, negative predictive value; *SENS*, sensitivity; *SPEC*, specificity; *F1*, a weighted average of the PPV and SENS

### HR-OI risk score (HR-OIRS)

**Fig. 4:**
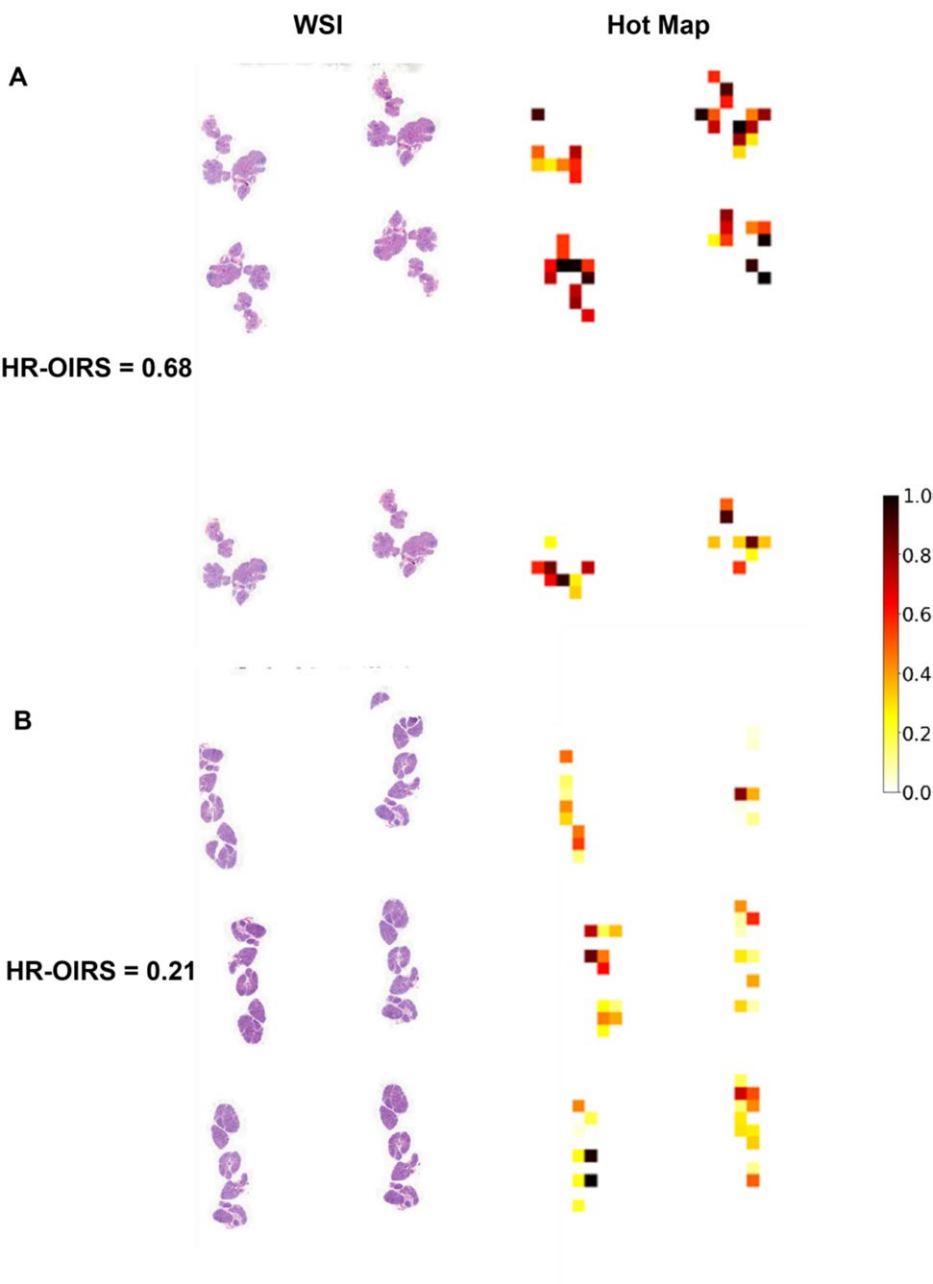
The visualized WSI hot maps provide not only the calculated HR-OIRS but also a visual representation of the relative importance of each patch. **A** For the WSI of a patient with HR-OI, the HR-OIRS is 0.68, and most patches are colored in darker shades of red, corresponding to the high-risk region on the color bar. **B** For the WSI of a patient without HR-OI, the HR-OIRS is 0.21, and most patches are colored in lighter shades of yellow, corresponding to the low-risk region

Figure 4 presents the visualized WSIs of patients, providing not only the calculated HR-OIRS but also a visual representation of the relative importance of each patch. Specifically, for the WSI of a patient with HR-OI (Fig. 4A), the HR-OIRS is 0.68, and most patches are colored in darker shades of red, corresponding to the high-risk region on the color bar. In contrast, for the WSI of a patient without HR-OI (Fig. 4B), the HR-OIRS is 0.21, and most patches are colored in lighter shades of yellow, corresponding to the low-risk region.

### Morphological differences

By carefully comparing patches from the low-risk and high-risk groups, we can identify morphological differences that may contribute to the differential risk assessment. Patients with HR-OI demonstrated more severe lymphocytic, plasmacytic, or eosinophilic infiltrates. These inflammatory cells were more frequently observed surrounding acini or ducts, or even infiltrating into the acinar parenchyma. Additionally, a subset of patients exhibited more pronounced acinar atrophy, degeneration, or necrosis, along with more severe interstitial fibrosis.

## Discussion

This study employed AI techniques to model and analyze WSIs of labial gland biopsies from patients with SS, with the aim of predicting the occurrence of HR-OI. Unlike previous work that bundled heterogeneous complications, we focused on HR-OI because these lesions drive irreversible damage and long-term mortality in SS. At the clinically chosen cutoff (HR-OIRS ≥ 0.50), the model correctly classified 74% of patients in the external cohort. A sensitivity of 0.73 means that almost three-quarters of patients with forthcoming major-organ involvement were detected, while a specificity of 0.73 limits over-referral. The 67% PPV and 79% NPV provide clinicians with actionable probabilities for shared decision-making. Our findings have yielded several innovative contributions.

Firstly, traditional pathological image analysis relies heavily on the experience and subjective judgment of pathologists, often leading to low efficiency and potential errors. Our RAIPSS model can automatically analyze WSIs, providing objective and quantitative results, thereby significantly improving analysis efficiency and accuracy. While traditional prognostic assessment for SS primarily relies on clinical symptoms and laboratory tests, these markers often exhibit limited specificity and sensitivity. Our RAIPSS model can predict the risk of HR-OI in SS patients, offering a novel reference for prognostic evaluation.

Secondly, through AI-based analysis of SS patient WSIs, we identified a strong correlation between certain WSI features and the risk of HR-OI. For instance, features such as lymphocytic infiltration, acinar destruction, and fibrosis were positively correlated with the risk of HR-OI in SS patients. These features can serve as early warning indicators of HR-OI in SS patients.

Thirdly, our findings provide new insights into the treatment and management of SS patients. By predicting the risk of HR-OI in SS patients using our RAIPSS model, high-risk patients can be identified early, enabling the implementation of effective preventive measures to reduce the incidence of HR-OI. Additionally, personalized follow-up and imaging schedules can be developed based on the WSI features and HR-OI risk of individual patients, leading to improved treatment outcomes.

Fourthly, our study, which utilizes AI to analyze SS patient WSIs, offers novel perspectives and methodologies for SS pathological research. Future studies can further explore the relationship between other WSI features and disease progression in SS patients, and develop more precise AI models to provide better diagnostic and therapeutic support for SS patients.

Although our study has achieved some innovative results, there are still limitations that need to be addressed in future research.

First, because of the cross-sectional design, longitudinal follow-up data were not available; therefore, we could not evaluate the temporal evolution of HR-OI or the model’s ability to predict future organ damage. A prospective, multicentre cohort with serial imaging and outcome ascertainment is currently being planned to address this gap.

Secondly, the sample size of our study is relatively small, which may limit the generalizability of our model. Future studies should expand the sample size to improve the model’s generalizability.

Thirdly, our study only included biopsy specimens. Future studies could explore other imaging data, such as ultrasound and CT, in combination with WSI data to construct more multidimensional AI models.

Additionally, because our cohort comprised only newly diagnosed, untreated patients, the study could not evaluate how immunomodulatory therapy might alter HR-OI risk or modify the model’s performance. A prospective follow-up study—including serial imaging and recorded treatment courses—is underway to address this limitation.

Finally, the application of AI models in the medical field raises ethical concerns, such as data privacy and fairness. Future research should consider these ethical issues and develop appropriate ethical guidelines to ensure the safe and compliant use of AI models.

## Conclusion

This study established a RAIPSS model based on WSIs of labial gland biopsies from SS patients and evaluated its ability to predict HR-OI in these patients. Our results demonstrate that the model can automatically identify key features within WSIs and correlate them with the risk of OI. Furthermore, we discovered a correlation between specific WSI features in SS patients and their risk of OI. For instance, features such as lymphocytic infiltration, acinar destruction, and fibrosis were positively associated with the risk of OI in SS patients. These findings suggest that AI technology can be applied to analyze WSIs of labial salivary gland biopsies in SS patients, predict the risk of OI, and provide a novel reference for prognostic evaluation in SS patients.

## Supplementary Information

Below is the link to the electronic supplementary material.

Supplementary file1 (DOCX 15 KB)

## Data Availability

The original contributions presented in the study are included in the article/Supplementary Material. Further inquiries can be directed to the corresponding authors.

## References

[1] Qin B, Wang J, Yang Z et al (2015) Epidemiology of primary Sjögren’s syndrome: a systematic review and meta-analysis [J]. Ann Rheum Dis 74(11):1983–1989 24938285 10.1136/annrheumdis-2014-205375

[2] Maciel G, Crowson C, Matteson E et al (2017) Incidence and mortality of physician-diagnosed primary Sjögren syndrome: time trends over a 40-year period in a population-based US cohort [J]. Mayo Clin Proc 92(5):734–743 28389066 10.1016/j.mayocp.2017.01.020 PMC5470777

[3] Negrini S, Emmi G, Greco M et al (2022) Sjögren’s syndrome: a systemic autoimmune disease [J]. Clin Exp Med 22(1):9–25 34100160 10.1007/s10238-021-00728-6 PMC8863725

[4] Baldini C, Pepe P, Quartuccio L et al (2014) Primary Sjogren’s syndrome as a multi-organ disease: impact of the serological profile on the clinical presentation of the disease in a large cohort of Italian patients [J]. Rheumatology (Oxford) 53(5):839–844 24369420 10.1093/rheumatology/ket427

[5] Prak R, Arends S, Verstappen G, et al (2022) Fatigue in primary Sjögren’s syndrome is associated with an objective decline in physical performance, pain and depression [J]. Clin Exper Rheumatol. 10.55563/clinexprheumatol/70s6cs 36226629

[6] Ioannidis JP, Vassiliou VA, Moutsopoulos HM (2002Mar) Long-term risk of mortality and lymphoproliferative disease and predictive classification of primary Sjögren’s syndrome. Arthritis Rheum 46(3):741–747 11920410 10.1002/art.10221

[7] Huang H, Xie W, Geng Y et al (2021) Mortality in patients with primary Sjogren’s syndrome: a systematic review and meta-analysis [J]. Rheumatology (Oxford) 60(9):4029–4038 33878179 10.1093/rheumatology/keab364

[8] Singh A, Singh S, Matteson E (2016) Rate, risk factors and causes of mortality in patients with Sjögren’s syndrome: a systematic review and meta-analysis of cohort studies [J]. Rheumatology (Oxford) 55(3):450–460 26412810 10.1093/rheumatology/kev354 PMC5009445

[9] Yazisiz V, Göçer M, Erbasan F et al (2020) Survival analysis of patients with Sjögren’s syndrome in Turkey: a tertiary hospital-based study [J]. Clin Rheumatol 39(1):233–241 31555987 10.1007/s10067-019-04744-6

[10] Goules AV, Tatouli IP, Moutsopoulos HM et al (2013) Clinically significant renal involvement in primary Sjögren’s syndrome: clinical presentation and outcome. Arthritis Rheum 65:2945–2953 24166794 10.1002/art.38100

[11] Currie G, Hawk KE, Rohren E, Vial A, Klein R (2019Dec) Machine learning and deep learning in medical imaging: intelligent imaging. J Med Imaging Radiat Sci 50(4):477–487 31601480 10.1016/j.jmir.2019.09.005

[12] Palm O, Garen T, Berge Enger T et al (2013Jan) Clinical pulmonary involvement in primary Sjogren’s syndrome: prevalence, quality of life and mortality–a retrospective study based on registry data. Rheumatology (Oxford) 52(1):173–179 23192906 10.1093/rheumatology/kes311

[13] Fauchais A-L, Ouattara B, Gondran G et al (2010Jun) Articular manifestations in primary Sjögren’s syndrome: clinical significance and prognosis of 188 patients. Rheumatology (Oxford) 49(6):1164–1172 20299380 10.1093/rheumatology/keq047

[14] Palm O, Garen T, Berge Enger T et al (2013) Clinical pulmonary involvement in primary Sjogren’s syndrome: prevalence, quality of life and mortality–a retrospective study based on registry data [J]. Rheumatology (Oxford) 52(1):173–179 23192906 10.1093/rheumatology/kes311

[15] Gao H, Zhang X, He J et al (2018) Prevalence, risk factors, and prognosis of interstitial lung disease in a large cohort of Chinese primary Sjögren syndrome patients: A case-control study [J]. Medicine 97(24):e11003 29901591 10.1097/MD.0000000000011003 PMC6023797

[16] Brito-Zerón P, Kostov B, Solans R et al (2016) Systemic activity and mortality in primary Sjögren syndrome: predicting survival using the EULAR-SS Disease Activity Index (ESSDAI) in 1045 patients [J]. Ann Rheum Dis 75(2):348–355 25433020 10.1136/annrheumdis-2014-206418

[17] Zhong H, Liu S, Wang Y et al (2022) Primary Sjögren’s syndrome is associated with increased risk of malignancies besides lymphoma: a systematic review and meta-analysis [J]. Autoimmun Rev 21(5):103084 35341972 10.1016/j.autrev.2022.103084

[18] Retamozo S, Brito-Zerón P, Ramos-Casals M (2019) Prognostic markers of lymphoma development in primary Sjögren syndrome [J]. Lupus 28(8):923–936 31215845 10.1177/0961203319857132

[19] Alesini G, Priori R, Bavoillot D et al (1997) Differential risk of non-Hodgkin’s lymphoma in Italian patients with primary Sjögren’s syndrome [J]. J Rheumatol 24(12):2376–2380 9415645

[20] Liang Y, Yang Z, Qin B, Zhong R (2014) Primary Sjogren’s syndrome and malignancy risk: a systematic review and meta-analysis. Ann Rheum Dis 73:1151–1156 23687261 10.1136/annrheumdis-2013-203305

[21] Salomonsson S, Jonsson MV, Skar-Stein K et al (2003) Cellular basis of ectopic germinal center formation and autoantibody production in the target organ of patients with Sjögren’s syndrome. Arthritis Rheum 48:3187–201 14613282 10.1002/art.11311

[22] LeCun Y, Bengio Y, Hinton G (2015) Deep learning. Nature 521:436–444 26017442 10.1038/nature14539

[23] van der Laak J, Litjens G, Ciompi F (2021) Deep learning in histopathology: the path to the clinic. Nat Med 27:775–784 33990804 10.1038/s41591-021-01343-4

[24] Skrede OJ, De Raedt S, Kleppe A et al (2020) Deep learning for prediction of colorectal cancer outcome: a discovery and validation study. Lancet 395:350–360 32007170 10.1016/S0140-6736(19)32998-8

[25] Coudray N, Ocampo PS, Sakellaropoulos T et al (2018) Classification and mutation prediction from non-small cell lung cancer histopathology images using deep learning. Nat Med 24:1559–1567 30224757 10.1038/s41591-018-0177-5 PMC9847512

[26] Pantanowitz L, Quiroga-Garza GM, Bien L et al (2020) An artificial intelligence algorithm for prostate cancer diagnosis in whole slide images of core needle biopsies: a blinded clinical validation and deployment study. Lancet Digit Health 2:e407–e416 33328045 10.1016/S2589-7500(20)30159-X

[27] Courtiol P, Maussion C, Moarii M et al (2019) Deep learning-based classification of mesothelioma improves prediction of patient outcome. Nat Med 25:1519–1525 31591589 10.1038/s41591-019-0583-3

[28] Chen CL, Chen CC, Yu WH et al (2021) An annotation-free wholeslide training approach to pathological classification of lung cancer types using deep learning. Nat Commun 12:1193 33608558 10.1038/s41467-021-21467-y PMC7896045

[29] EhteshamiBejnordi B, Veta M, Johannes van Diest P et al (2017) Diagnostic assessment of deep learning algorithms for detection of lymph node metastases in women with breast cancer. JAMA 318:2199–2210 29234806 10.1001/jama.2017.14585 PMC5820737

[30] Wang X, Chen Y, Gao Y et al (2021) Predicting gastric cancer outcome from resected lymph node histopathology images using deep learning. Nat Commun 12:1637 33712598 10.1038/s41467-021-21674-7 PMC7954798

[31] Shi JY, Wang X, Ding GY et al (2021) Exploring prognostic indicators in the pathological images of hepatocellular carcinoma based on deep learning. Gut 70:951–961 32998878 10.1136/gutjnl-2020-320930

[32] Kermany DS, Goldbaum M, Cai W et al (2018) Identifying medical diagnoses and treatable diseases by image-based deep learning. Cell 172(5):1122-1131.e1129 29474911 10.1016/j.cell.2018.02.010

