# Supplementary material for: Artificial intelligence-based prediction of organ involvement in Sjogren’s syndrome using labial gland biopsy whole-slide images
Source: Clin Rheumatol. 2025 Jun 5;44(7):2919–27. doi: 10.1007/s10067-025-07518-5 (PMC12234637; doi:10.1007/s10067-025-07518-5)
Supplement: Supplementary file 1 — Supplementary file1 (DOCX 15 KB) [file 10067_2025_7518_MOESM1_ESM.docx]

**Artificial Intelligence-Based Prediction of Organ Involvement in Sjogren's Syndrome Using Labial Gland Biopsy Whole Slide Images**

**Methodological supplements：**

**WSI Data Preprocessing.** Labial gland biopsy specimens were collected under aseptic conditions, fixed, embedded, and stained with hematoxylin and eosin (H&E). Subsequently, high-quality whole slide imaging scanners were used to scan the specimens at a resolution of 40x magnification[23-27]. Dataset 1 was scanned using a KF-PRO-020 scanner (KONFOONG Biotech, China), while dataset 2 was scanned using a Panoramic 250 FLASH scanner (3DHISTECH Ltd, Budapest, Hungary). All scanned images were stored in a secure database under a unified naming convention. All images underwent quality control to ensure clarity, contrast, and focus. Due to the potentially enormous size of whole slide images, containing hundreds of millions of pixels, exceeding the processing capabilities of typical computers or graphics processing units (GPUs), processing the entire image can be challenging. Using the OpenSlide library, the WSI was sampled at the maximum scan resolution with a non-overlapping sliding window size of 512x512 pixels to generate a series of patches, while saving the original positional coordinates of each patch on the WSI for reconstruction. Patches containing no tissue cells, or those that were blurry or unclear due to defocus, stains, or other reasons, were removed. In dataset 1, 80% of the samples were randomly selected as the training dataset for model training, and the remaining 20% were used as the internal test dataset. All samples in dataset 2 were used as the external test dataset.

**Patch-Based Feature Extractor.** Each segmented patch requires feature extraction to enable subsequent model understanding and processing. We used three different pre-trained convolutional neural network (CNN) models as feature extractors[22]: ResNet50, InceptionV3, and EfficientNet-B5. These models have been pre-trained on large-scale image datasets such as ImageNet and can capture rich image features.

ResNet50 Feature Extractor. ResNet50 is a classic deep residual network that addresses the vanishing gradient problem in deep neural networks by introducing residual connections. We removed the final fully connected layer of ResNet50 and retained only the convolutional layers. These convolutional layers are responsible for extracting high-dimensional feature representations of patches. After processing by ResNet50, each patch generates a 2048-dimensional feature vector.

InceptionV3 Feature Extractor. InceptionV3 employs Inception modules, enabling efficient extraction of image features at different scales. Similarly, we removed the classification layer of InceptionV3 and retained the part used for feature extraction. Due to its design considering multi-scale information, InceptionV3 can extract richer features. After processing by InceptionV3, each patch generates a 2048-dimensional feature vector.

EfficientNet-B5 Feature Extractor. EfficientNet is a family of models optimized through neural architecture search, achieving a good balance between computational efficiency and accuracy. EfficientNet-B5 is one of its variants and has strong feature extraction capabilities. We used its feature extraction part and removed the classification layer. After processing by EfficientNet-B5, each patch generates a 2048-dimensional feature vector.

**Multi-Instance Learning (MIL) Model Construction.** Within the MIL framework, each WSI is considered as a bag containing multiple patches (instances). Our task is to classify the entire WSI, thus requiring the extraction of global information from the features of these patches.

Vision Transformer (ViT) Model. We constructed a separate ViT model for each feature extractor. ViT is a model based on the self-attention mechanism, originally designed for natural language processing tasks, and later extended to image processing. ViT classifies images by dividing them into multiple patches, encoding each patch as a vector, and then processing the relationships between these vectors. Input to the ViT model: The feature vectors (2048-dimensional) of each patch in a WSI are input into the ViT model. For all patches of a WSI, these features are stacked into a two-dimensional tensor. Self-attention mechanism of ViT model: The ViT model captures the relationships and global information between patches through a multi-head self-attention mechanism. The self-attention mechanism can calculate the importance of each patch based on the features of all patches and dynamically adjust the feature representation of each patch. Feature aggregation: The output of the ViT model is the feature vector of each patch. These vectors are aggregated (using average pooling) to obtain a global feature representation of the entire WSI. This representation reflects the overall information of the WSI and can be used for subsequent classification.

After obtaining the global features of the WSI, the ViT model outputs a binary classification probability value through a linear classifier. This probability value represents the likelihood of the WSI belonging to a certain class. Each feature extractor (ResNet50, InceptionV3, EfficientNet-B5) corresponds to an independent ViT model, so we finally obtain three independent binary classification models.

**Ensemble Model Risk Prediction and RAIPSS.** To improve predictive performance and robustness, an ensemble learning strategy was employed to fuse the prediction results of the three multi-instance models by calculating the arithmetic mean[34]. The resulting value was defined as the HR‑OI risk score (HR‑OIRS) for each patient, ranging from 0 to 1. A lower HR‑OIRS indicates a lower probability of developing HR‑OI, while a higher HR‑OIRS suggests a higher risk. We refer to this series of algorithms, which first uses CNN models for initial patch feature extraction, then uses attention-based ViT models for further modeling and analysis of whole-slide image features, and finally employs an ensemble algorithm to fuse the output and obtain the final predicted risk probability, as the Risk prediction Artificial Intelligence model for HR‑OI of Sjogren's syndrome (RAIPSS). RAIPSS can significantly improve the stability and reliability of the final model, reducing the risk of bias associated with a single model.

**Whole Slide Image Interpretability.** After applying multi-instance learning for modeling, we can not only obtain the classification prediction result for the entire WSI (presence or absence of HR‑OI) but also acquire the contribution of each patch instance to the final prediction result, thereby identifying the most critical pathological slice regions for the classification task. During model inference, when inputting a WSI dataset, the model calculates an attention weight score for each patch instance, allowing us to analyze the key slice regions that have the greatest impact on the prediction result. By normalizing the weight scores to the range of (0, 1) and visualizing them using different color bars, these regions are likely to contain important pathological features, providing intuitive and valuable interpretations for clinical diagnosis and complication risk assessment.

Evaluation Methods. To comprehensively evaluate the predictive performance of the model, we employed the following statistical analysis methods: We used several statistical metrics to assess the model's performance. By comparing the model's predicted positive/negative results with the true labels, we can calculate the proportions of true positives (TP), true negatives (TN), false positives (FP), and false negatives (FN), and obtain metrics such as accuracy, sensitivity, and specificity. The ROC curve plots the true positive rate (TPR) and false positive rate (FPR) of the model at different thresholds. The area under the ROC curve (AUC) reflects the overall predictive power of the model. The AUC value ranges from 0 to 1, with higher values indicating better predictive performance. The F1-score is the harmonic mean of precision and recall, also ranging from 0 to 1, with higher values indicating better performance. We primarily evaluated the model based on the AUC and F1-score, while also considering other metrics. We used Openslide to process WSIs and generate patches, built the artificial intelligence model using TensorFlow, and performed data analysis and statistical calculations using Python.
